# Beginning a Partnership with PhotoVoice to Explore Environmental Health and Health Inequities in Minority Communities

**DOI:** 10.3390/ijerph111111132

**Published:** 2014-10-27

**Authors:** Melinda Butsch Kovacic, Sara Stigler, Angela Smith, Alexis Kidd, Lisa M. Vaughn

**Affiliations:** 1 Cincinnati Children’s Hospital Medical Center, Department of Pediatrics, University of Cincinnati, 3333 Burnet Avenue, Cincinnati, OH 45229, USA; E-Mails: sara.stigler@ucmail.uc.edu (S.S.); lisa.vaughn@cchmc.org (L.M.V.); 2 Seven Hills Neighborhood Houses Findlay Street Center, 901 Findlay Street, Cincinnati, OH 45214, USA; E-Mails: radka49@gmail.com (A.S.); miss_kidd@yahoo.com (A.K.)

**Keywords:** environmental health, health inequities, community health, PhotoVoice, child/adolescent health, community-based participatory research, community engagement, minority health, health disparities

## Abstract

Research informs action, but the challenge is its translation into practice. The 2012–2017 National Institute of Environmental Health Sciences Strategic Plan emphasizes partnership with community stakeholders to capture critical missing information about the effects of environment on health and to improve translation of study results, a daunting task for many traditionally-trained researchers. To better understand economic and neighborhood context consistent with these goals as well as existing inequities, we needed access to a highly affected community to inform and participate in our research. Our team therefore undertook a PhotoVoice project as a first step in establishing a participatory partnership and to appreciate the lived experiences of and build trust with youth visiting an urban community center in a high-risk, low-income, African American neighborhood located along a busy, polluted interstate. Ten 8–13 years-olds represented their community’s perspectives through photographs over 14-weeks using structured questioning. Five themes emerged: poor eating habits/inadequate nutrition; safety/violence; family/friends/community support; future hopes/dreams; and garbage/environment. Public viewings of the photos/captions facilitated engagement of other community agencies and multidisciplinary academic faculties to work together to build a sustainable “community collaboratory” that will promote health at the center by providing families knowledge/skills to prevent/minimize environmental exposures via diet/lifestyle changes using community-engaged, citizen scientist and systems thinking approaches.

## 1. Introduction

One purpose of research is to inform action or produce knowledge that can be translated to improve health or influence policy outside of the research project [1]. One problem that often plagues progress in health-related research is its slow translation into practice. More than often, researchers are not well-positioned to implement their research findings. The 2012–2017 Strategic Plan for the National Institute of Environmental Health Sciences (NIEHS) includes a set of descriptive strategic themes as well as a collection of 11 specific strategic goals that focus on how low level, common exposures contribute to the development of widespread disorders like asthma and obesity among others [2]. Broadly, the plan emphasizes the need for observational population-based, translational and systems-based or “omic” approaches in confirming laboratory-based findings while recognizing the totality of a person’s environmental exposures or “exposome” from all sources and routes, across the life span; and specifically stresses the critical need for interdisciplinary collaborations and partnerships with stakeholders and communities, particularly those that are socioeconomically disadvantaged and greatly suffer inequalities in both health and environmental burdens [2]. 

For many traditionally trained researchers, even epidemiologists and clinical researchers already accustomed to working with data from populations, such a push is largely overwhelming or at least, uncomfortable. To entice and assist researchers, the NIEHS, the National Institute of Minority Health and Health Disparities (NIMHD) and other institutes at the National Institutes of Health (NIH) have provided an increasing number of training programs and funding applications that require transdisciplinary, community engagement and translational research, the three pillars of third generation research that is believed necessary to achieve health equity for all [3]. The Clinical Translational Science Awards (CTSAs), for example, include elements focusing on community engagement [4]. The NIMHD Comprehensive Centers of Excellence programs for research require collaboration with established partnerships with community based organizations. Further, special review panels and an NIH-wide Interest Groups (including the National Heart, Lung and Blood Institute) have been initiated to increase awareness, career development, use, and funding of translational and more specifically, community-engaged or community-based participatory research (CBPR). CBPR is a collaborative approach or orientation to conducting research that involves all partners, particularly those most affected by the research, in the research process. As a result researchers are challenged to listen to, learn from, solicit and respect the contributions of, and share power, information, and credit for accomplishments with groups or communities that they are targeting [5]. Thought to be the ideal by some, it is at the right side of the community engagement continuum [6].

PhotoVoice is a participatory method where participants are asked to represent their community or perspectives through photographs in order to capture information about issues of importance to them [7]. Coming out of CBPR as well as participatory action research (PAR), PhotoVoice may assist researchers in developing a relationship with community to initiate CBPR in the future by helping them to better understand community member’s lived experiences. The approach is increasingly being utilized to study health disparities and health outcomes in both youth and adults likely because of the need to consider many different personal and community factors [8]. Important to the methodology are the often deep discussions surrounding the photographs [9], their accompanying narratives, and subsequently, the education, outreach and action taken as a result of the collaborative research project. The approach can also serve health researchers wishing to partner with vulnerable or medically underserved populations as both a needs assessment and tool to begin to build trust in the community. It helps researchers to acknowledge what the community thinks is important for their health, prioritize and plan respectfully. The use of images helps participants, particularly children, not only to creatively and effectively realize their opinions on difficult subjects, but also to better share them in a supportive environment [9,10]. There are tangible and immediate benefits to participants as well. Given their newly rediscovered understanding of their health or community, previous studies suggest that child and youth participants of PhotoVoice are better educated about community health, often feel more empowered to take action, and/or are more likely to encourage and participate in health promotion behaviors as well as in future research studies [9,10]. 

The objective of this PhotoVoice project was to gain important perspectives anchored by lived experiences of youth utilizing a neighborhood community center in Cincinnati, Ohio’s West End neighborhood, USA. In understanding their lived experiences, completion of the project would help investigators at Cincinnati Children’s Hospital Medical Center to better partner with both the youth and the center staff to better tailor future health education programs and health research projects focused on both infectious and chronic disease prevention and treatment. Specifically, we were interested in understanding how minority children living in low-income neighborhoods perceive the influence of the environment on their health, and how we (as academic partners) could improve health education and future health outcomes given the exposures and lifestyles present. There are few opportunities for these children to share their perceptions on a broader scale. 

Indeed, nearly 88% of the population living in the West End Neighborhood are African American; 56.9% are female; 25.4% of residents are younger than 20 years old; and only 24.2% of adults aged 25 years and older have a degree beyond high school [11]. Approximately 70% of all households are single mother households compared to 27.5% of households in Cincinnati [12]. Further, the neighborhood is located along the highly traveled interstate highway corridor. Residents are exposed to high levels of traffic-related air pollution in addition to pollution from numerous manufacturing plants [13]. The area is essentially a food desert and while there is a nearby medical clinic, residents, upon informal interview, indicated that it had not adequately replaced the multi-purpose primary care facility that it supplanted in 2010. Among all the Cincinnati neighborhoods, the life expectancy of residents is 69.8 years [14], 18 years less than those living in the most affluent neighborhood in the larger metropolitan area. As observed gaps in life expectancies are likely tied to the incidence of chronic disease [3], we desired a community partnership to facilitate mutually beneficial research targeting understanding associations between exposures and chronic disease.

## 2. Methods

### 2.1. Partnering Community Center 

Our community partner, the Seven Hills Neighborhood Houses Findlay Street Center, is a one-stop social service agency and community center historically offering a wide array of client-driven services to local residents. The center seeks to provide an opportunity for less privileged children to improve their chances in life by breaking the cycle of poverty that has existed in this community for decades understanding that with poverty, there are greater chances of poor nutrition, obesity, lifelong chronic disease and limited access to quality education and financial opportunities. Indeed, most (74%) of the center’s clients have annual incomes less than $15,000 and most (92%) are African American. Clients include children (32%), young adults (32%), and older adults, aged >55 (36%). 

Founded in 1961, the center’s mission has been “As a partner in the communities we serve, we are dedicated to improving the quality of life of our neighbors. To this end, this mission has historically been fulfilled by actively partnering with area schools, organizations, businesses, community members, and universities to provide strategic programming and services that are tailored specifically for respective community member groups. These collaborations have also allowed the center to maximize their ability to serve, increase their resources, and reduce their costs. Programs are client driven and have included victims of crime advocacy and counseling, senior wellness and support, academic assistance, visual and performing arts, after school care, summer activities, and athletics. Child and youth initiatives have typically been preventative in nature, and include activities and programs intended to break the cycle of poverty. Extensive sports programs are offered. In addition, the nutritional needs of clients are met via an Emergency Food Pantry, Daily Bread Room, and Congregate Meal Programs. Dedicated youth and social service professionals, many of whom have been with the agency for many years, volunteer to help facilitate the center’s mission. Indeed, the center is one of the few organizations still standing and serving the poorest families living in the neighborhood. 

Within the past few years, three of the area’s schools, a longstanding local health clinic, two restaurants, and several stores and businesses have closed. The families living within their service area have not experienced the revitalization of their homes like nearby areas have. The center regularly holds events that foster family involvement (e.g., Back to School Hair-a-thon, Boxing Shows, Tea Parties, Family Community Nights, and an End of Summer Celebration). In recent years, families have increasingly voiced concerns about their growing healthcare needs and about the future health of their children. Importantly, at a community forum, a number of residents indicated that they were concerned about air pollution and respiratory diseases (emphysema and asthma), poor nutrition and obesity, high blood pressure, addiction and mental illness. 

### 2.2. Participant Selection and Consent 

After active involvement in community activities and providing educational programming over a six-month period, investigators approached staff at the center about participation in a PhotoVoice project. Enthusiastic, the center staff invited researchers to give an overview to possible participants during their After School Program. The overview consisted of an explanation of health (Table 1) defined broadly as an ever-changing process of trying to achieve one’s own potential with respect to body, heart, mind, and spirit. Special emphasis was placed on how the environment in which the children live influences health in all 4 areas. Further, the presentation covered how photography could be used to better understand how they feel about their health. More than thirty children attended. After the presentation, children self-identified as possible participants (N = 16). The center staff approved the participation of 12 of these children excluding a single child because of past extremely aggressive and confrontational behavior and 3 children because of limited prior program participation and inability to commit to our participation requirements of at least 4 sessions completed. A written description of the study approved by the Cincinnati Children’s Hospital Medical Center Institutional Review Board (IRB) was sent home with each child. A single center staff member was trained in how to appropriately obtain informed parental permission from parents and caregivers. Over a three-week period, center and study staff contacted and obtained verbal parental permission from the parents of 10 of these children (waiver of documentation of parental permission approved by our IRB) after explaining the study, risks and benefits of the study either in person or by phone. At our first meeting, study staff then further explained the requirements and expectations of the PhotoVoice project to the participating group and each child provided assent. Written parental permission/consent was obtained to include a single photo that includes a child’s image in this report. Brief demographic information was collected at the beginning of the project to describe the study population sample. 

**Table 1:** Areas of health and PhotoVoice lessons taught.

Health Area	Definition	Related Factors
Body	The ability to physically perform normal activities of daily living.	Having good hygiene, nutrition, active care of illnesses, and being physically fit.
Heart	The importance of promoting positive actions that build self-esteem, self-confidence, understanding of appropriate feelings, and what builds a good relationship with others.	Appropriate use of social supports and friendships; the ability to adapt to various social situations.
Mind	Having the ability to think clearly and to reason objectively; to learn and use what is learned appropriately.	Encourage continued learning and being prepared to use “brain power” effectively to meet various challenges.
Spirit	The ability of having hope and purpose in life that involves various attributes of values, attitudes, and belief structures.	Understanding one’s own ability to make a positive contribution in the community and the world.
Environment	Understanding the effects of the larger environment on our health and the role we all play to preserve, protect, and improve environmental conditions.	The effects of pollution of land, air, water, and food supply; over-reliance of non-renewable energy sources; overproduction of waste; exploitation of natural resources.

### 2.3. Setting of PhotoVoice

The primary location of the PhotoVoice sessions occurred in either the library or computer room at the partnering center. In addition, two field trips were taken, each with 5 children attending. The first trip was a walking tour of their nearby community. A second trip was a driving tour of the larger neighborhood including the adjacent industrial and retail areas. The drive ended at the local children’s museum where the children toured the science museum and attended an environmentally inspiring film.

### 2.4. Study Procedures 

After giving assent, participants were reminded about the broad definition of health and given training on the research methodology, their responsibilities, and protection of privacy. Next, there was a discussion on proper use of cameras for the purpose of the study. Each participant was then provided a 7 MP Vivitar V7122 Digital Camera with a 4-megabyte secure digital (SD) card and their first photographic assignment. That week, and each meeting thereafter, children were to target one of the areas of health (*i.e.*, heart, mind, body and spirit) selected by the larger group. Facilitators assisted the participants in understanding their assignments by providing examples and making suggestions, but ultimately, the participants themselves decided on what to photograph. At the start of the following meeting, study staff would collect the SD cards and upload the photographs, renaming them with the child’s specific identifier. Participants would then engage in a photography discussion session where children reflected on their photographs. The photographs were displayed by computer or by projector to the larger group. 

Each child would select 1 or 2 photos to discuss in more detail, using a structured questioning technique called SHOWED [15]. SHOWED stands for S: What do you SEE in the Photo? H: What is really HAPPENING? O: how does this relate to OUR lives? W: WHY does this situation, problem, or strength exist? D: what can I/we DO about it? To assist the children further, we encouraged them to exemplify their unique voices by adding an option for the letter E: express yourselves! Each child was also asked to identify all the health themes (body, mind, heart, spirit) associated with the photograph if any, and to provide a caption. At first, the children were asked to respond to these more detailed questions using pen and paper. However, as the literacy levels and writing skills of many of the children were lower than average, and because the task of writing was diminishing interest in the overall project, in subsequent weeks, following the larger group discussions, study staff members would assist participants one-on-one with the SHOWED questions (although the other participants were still in the same room), keeping the integrity of each response intact. Indeed, the answer response to each SHOWED question was transcribed word for word. If additional questions were asked, the answer responses were written word for word by the interviewees. The responses would then be reread to the participant to ensure that the responses were consistent with what had been said. The resulting responses were shared with the larger group. Often this approach led to even greater discussion, particularly in children that were more soft-spoken in the larger group. 

In total there were 14 weekly meetings associated with the PhotoVoice project (Table 2). However, photographic assignments were not given at each meeting and not all participants attended each week because of participation in other activities at school or at the center (*i.e.*, sports, dance *etc.*). These additional weeks allowed participants that had missed assignments to “catch up”. In addition, some meetings were reserved for social gatherings that included team and/or self-esteem building activities. As PhotoVoice typically ends with an action phase where participants organize and present to and/or share their photos with a broader community [9], participants and both study and center staff together identified and prepared for several public exhibitions at the center and in the community.

**Table 2:** Weekly PhotoVoice program agenda.

Week	Topic/Tasks
1	Overview and defined health (heart, mind, body, spirit)
2	SHOWED and photo caption instruction & practice
3	Camera instruction & practice; 1st assignment given: heart
4	Reviewed “heart” photos; SHOWED; 2nd assignment given: spirit
5	Team building exercises, SHOWED, assignment catch-up
6	Team building exercises; creation of rules for safe environment
7	Gallery Day planning, assignment catch-up
8	Reviewed “spirit” photos; SHOWED, 3nd assignment given: mind
9	Reviewed “mind” photos; SHOWED & caption writing
10	Walking neighborhood tour to take “body” photos
11	Reviewed neighborhood tour or “body” photos
12	SHOWED and caption writing; assignment catch-up
13	Driving tour and museum field trip
14	Driving tour and museum discussion; final SHOWED and caption writing; project wrap-up

### 2.5. Analysis 

Following each meeting, 3 study staff members reviewed their notes, any photographs received and the SHOWED responses both independently and together to identify broad themes. Specifically, staff selected and contextualized participants’ photos and sorted them by themes. From each group of photos, staff selected the images that they were most drawn to (of two or more images of the same object or person), verbalized the stories about what the photographs may mean, and then identified through written captions and SHOWED responses, important issues or themes. According to standard qualitative research procedure, all of the data were organized into broad conceptual categories and verified first among study staff and then among the participants at the beginning of each discussion in the following week for clarification, consensus, and credibility of important themes [16]. These themes were later confirmed in the final weeks of the project by encouraging participants to explore more broadly what was actually happening in select photos as well as other possible explanations or underlying meanings given the other themes. We considered participant photos, participant verbal and written reflections (when we had them), and our notes of group discussions of photos as well as discussions with center staff, select parents viewing the larger group of photos and other researchers to further establish credibility of themes [17]. Discussions often centered on whether or not the participants’ insights were similar across the larger groups of children frequently visiting the community center. 

### 2.6. Study Dissemination 

The study results were disseminated in by informal and formal presentations both with and without the participants present. Further, a colored newsletter was developed for broader dissemination of the study’s findings.

## 3. Results 

### 3.1. Study Participants 

In all, ten children ages 8–13 years participated (average age = 11.3 ± 1.6 years). All were African American with public health insurance. More than half were female (60%) and 90% were living with a single parent/guardian (seven mothers and one uncle). The remaining child lived with both parents (unmarried) at the beginning of the study, but with her grandmother and mother by the end of the study. All attended nearby Public Schools. None of the children were being treated for a chronic illness at the time of participation. Through more than 800 photographs taken and 14 total group meetings, 5 general themes were identified: (a) poor eating habits/inadequate nutrition; (b) safety/violence; (c) family/friends/community support; (d) future hopes/dreams; and (e) garbage/environment.

### 3.2. Body: Eating Habits and Nutrition 

**Figure 1:**
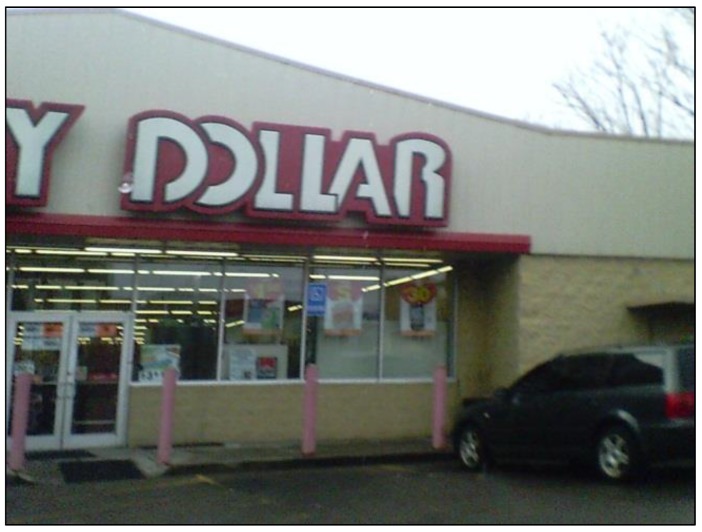
Photo caption: foods all about it; Health topic: body; “I go to Family Dollar when my momma feels like it. It sometimes is cheaper than Kroger, sometimes not. We get milk, cereal, juice, popcorn, and candy. We take the bus to the store so my momma doesn’t waste her gas. We sometimes go twice in a day since we can’t carry that much. I see a lot of people I know there. People can’t buy healthy food there. They want snacks. They are delicious. You don’t want people to think you don’t have candy every day. Everybody has candy after school, at school and at home. I eat a lot of candy. My family eats healthy sometimes but I am my own person. I tell them “don’t push my buttons and I won’t push yours”. All 4 of the kids in my family eat bad food and candy. We all talk back to our parents too”.

Several of the photographs and captions and accompanying discussions described healthy and unhealthy eating habits. In addition, the children debated how poor eating habits and failure to stay fit ultimately led to obesity and poor health in adulthood. One such photo caption (Figure 1) indicated that the child’s family frequently visited the local Family Dollar store by bus. The child points out that she likes to obtain candy at the store. While the child clearly acknowledges that candy is unhealthy, she also indicated she chose to eat it anyway despite her parents telling her not to. A second child’s photo of a nearby Big Boy restaurant described his realization that there were no healthy restaurants or chain grocery stores in his neighborhood. When further questioned about other places that sell food, he completely omitted the nearby historic, urban farmer’s market, a public market that sells meat, fish, poultry, locally grown fresh fruits and vegetables, cheese, deli, and ethnic foods located less than a mile away from the center (albeit outside of his immediate neighborhood). Interestingly, the market is a third party administrator of food stamps and accepts Ohio’s Women, Infants and Children (WIC) food assistance coupons enabling even low income residents to shop. When asked about the market, the boy indicated that he had rarely been there, and that he was uncertain why his family did not go there. Future studies are planned to more deeply explore usage of this market and other food providers in the area. 

### 3.3. Body and Spirit: Safety and Violence 

**Figure 2:**
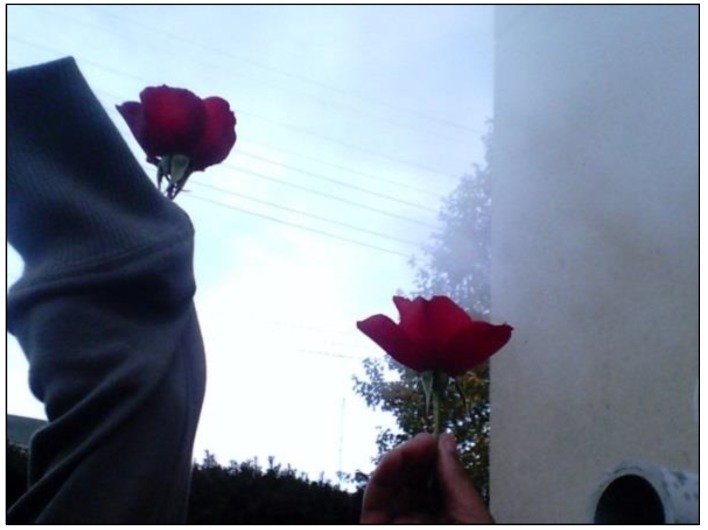
Photo caption: a day with destiny; Health topic: heart and soul; “This photo shows 2 flowers with steam in the background—the sun is shining through. It was cold outside and the flowers were frozen. We put them by steam from some pipes. The 2 flowers remind me of my cousins that died. It makes me think of my cousin who ran into another car while he was driving and he flew out and landed in a tree. The firemen kept looking for him for 7 h because they never looked up in the tree. His brother killed himself with a gun after that because he was tired of his family not listening to him. His girlfriend tried to help. I miss my cousin—I was his favorite. Even though they died, I think of flowers because I want to keep planting flowers and giving them to people I love”.

A number of the PhotoVoice participants suggested that safety and violence were issues in their community. The children discussed the presence of gangs in their neighborhood and why many kids had joined gangs to make money to live. Interestingly, the children empathized with gang members and recognized that education and strong family could help those in gangs. One child suggested that a stronger police force was needed. A second child took a photo of graffiti (stick couples holding hands next to stick children) on a building near the center. A man had been killed at that location the prior week. Even with this knowledge, the participant described how the graffiti symbolized the positive outlook of his family and community and how they helped him to stay healthy. Finally, a third child’s picture of two flowers (Figure 2) described her great loss associated with the tragic experiences of her two male cousins. Despite the many violent experiences, only on occasion was the concept of prayer mentioned by individual participants as a means to deal with the insecurities they felt and the violence they had experienced. However, prayer or faith was rarely discussed in the larger group as it seemed to be somewhat of a taboo topic.

### 3.4. Heart: Support of Family, Friends and the Community Center 

**Figure 3:**
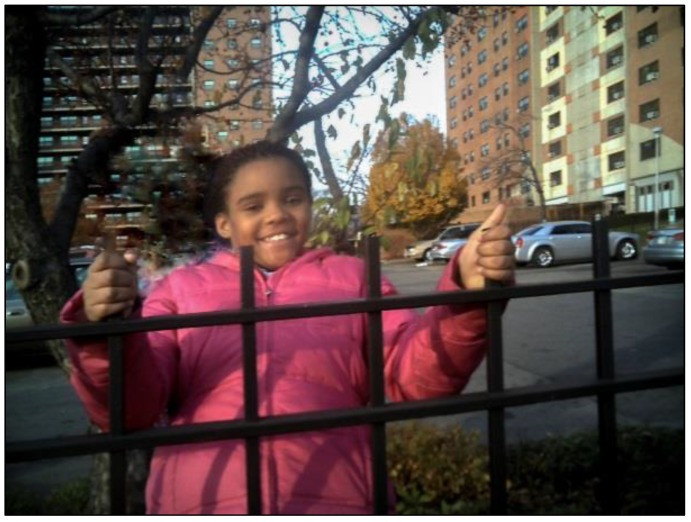
Photo caption: the real world; Health topic: heart; “My photo is of my friend. She is a real person. She doesn’t wear makeup. She is true to herself. She is creative and helps me when I am in trouble and helps me do my chores. This photo shows how she is enjoying herself with me and our friends. We were having fun. Friends are important because they help me and don’t say no…they really care for me. We can share secrets and trust each other. You can rely on a friend. I do not even have to pinkie promise with my friend. She is so strong. Lying to a friend shows they are not your true friend. Other people in my class lie and steel from me. Like my friend, I trust my mom too, but I haven’t seen my dad since I was 3 years old so I do not know if I can trust him”.

PhotoVoice participants discussed the importance of family members’ and friends’ roles in their own health. Children cited the importance of “being yourself” and both receiving and giving love as being critical to their relationships. One child mentioned that his family really wanted him to achieve success and to learn from their mistakes. Another photographed her best friend and described the characteristics of being a good friend (Figure 3). Raised by a single mom, the girl clearly found safety and hope in her closest advocates. On many occasions, the children indicated that they liked coming to the center (which they affectionately call the “Neighb”) to be with their friends and not have to deal with the stresses of life. Support of family and friends in fact was what most of the children felt helped them to continue to hope for a better future in “hard times”.

### 3.5. Mind: Future Hopes and Dreams 

**Figure 4:**
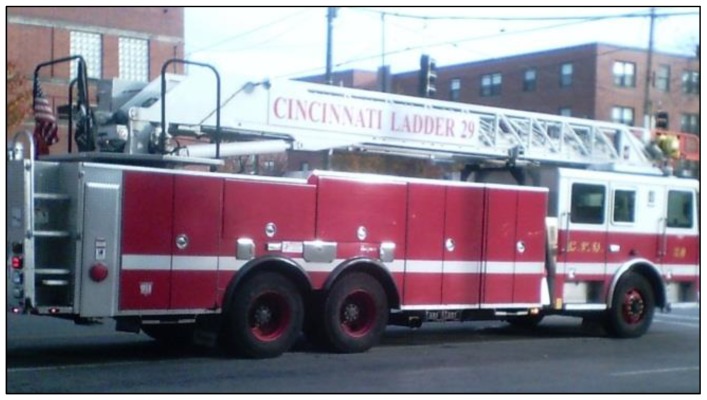
Photo caption: helpers; Health topic: body; “My photo is of a fire truck. We visited the fire station during PhotoVoice. Firemen use these trucks to rescue people. When people in my building have a problem, the firemen come. They can save lives. When I was 8 years old, I was in a house fire at my house. The stove was left on. The fireman came to put the fire out, but we had to get our clothes and move out. We stayed with my aunt. The fire scared me a little. The person that left the stove on was stupid. I was angry because I really loved that house. When I grow up, I want to be a doctor and save lives too. I need to stay in school and get good grades so that I can go to college and get a degree”.

A number of discussions focused on the future. The children clearly understood that continual learning, education and attending college could help them overcome challenges experienced by their neighbors and families. Still, while many identified what they wanted for their futures, few had specific plans to ensure that they would be successful. One boy indicated that he wanted to be a professional athlete, even though he was not a participant of any organized sport at his school or at the center. When asked about how he might achieve his goal, he understood that he would need practice and coaching, but was uncertain of how to begin to attain such assistance. Another child’s picture of a fire truck led to her description of her experience with a fire that destroyed her family’s home (Figure 4). As a result, she admired the fire fighters and explained that she too would like to serve her community in some way in her adult years. She indicated that she might want to be a police officer a doctor, but had not yet considered how she would reach her goal. 

### 3.6. Garbage and the Environment 

Interestingly, despite much education during the first meetings of the project on the topic by research staff, the environment and its effects rarely came up in early discussions. While there was evidence of pollution all around the center (*i.e.*, a discarded couch that had been discarded in an alley for weeks directly across from the center), the children did not photograph these items. When trash was pointed out to them, they often commented that they had not noticed it. With some discussion, several indicated that trash was everywhere and that it didn’t bother them. Instead of photographing litter, the children often photographed what they liked or wanted. For example, on a walking tour of the neighborhood, one child photographed and described a nearby newer building as “being pretty and clean compared to the old and dirty places” that she had lived all of her life. She suggested that she “wanted to live in a clean and pretty building when she grew up”. 

To help the children better understand the effects of the environment on their health, the group went on a driving tour of the larger neighborhood with their cameras in tow. For this tour, research and center staff alike questioned the children on their understanding of the buildings and other resources in their community and what roles they served. For example, upon passing a power plant, staff inquired about the resources required to make power for the city and the waste that remained as a result of making power. Upon passing a trash heap, staff again probed the children about their understanding of recycling and waste reduction. Several children indicated that their community would be better off if it were cleaner, but few indicated any motivation to help make it cleaner. The discussions continued as the group toured the local Children’s Science Museum, paying particular attention to the section on natural resources. 

**Figure 5:**
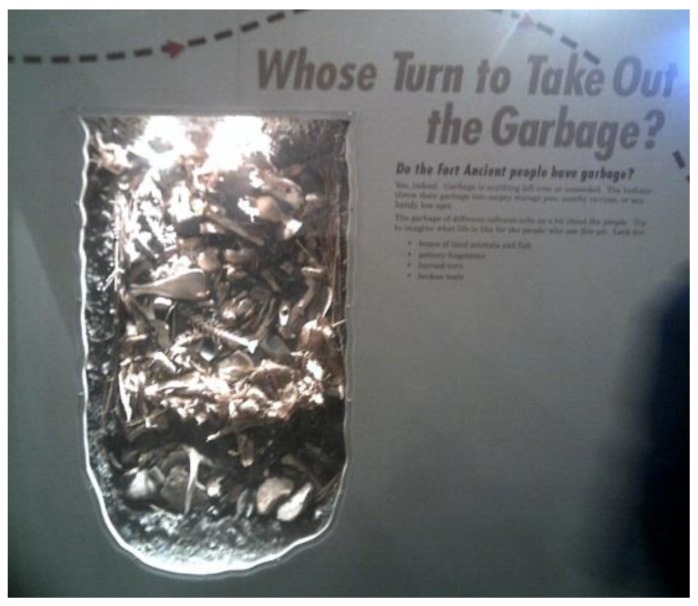
Photo caption: old garbage where it should be; Health topic: body and heart; “My photo shows garbage from long ago. There are animal bones, leaves, food remnants and dirt. In the ground, it is decomposing from all the years of being there. Today, people litter and put garbage everywhere on the ground around here instead of in the garbage can where it belongs. People are just lazy or don’t care. They don’t pick it up either. If you care about where you live, you wouldn’t do it and I don’t do it. To help, I can pick it up and put it in the trash. I can tell others not to litter. If I don’t let them do, maybe they won’t do it either. I don’t want to be lazy. I want to be successful and not disappoint my family by giving up my dreams. I want to be a NBA or NFL player. If there is a lot of garbage, it can pollute the air and that can affect your lungs. I need to be able to breathe and hold oxygen for long periods of time to play basketball and football. I need a plan and to practice more to reach my goal. I have not tried out for basketball yet because I have been afraid I will not make the team, but will try next school year”.

At the follow-up meeting a week after the tour, it was obvious that a few of the participants that had been on the tour had a better understanding of the effects of the environment on health. In fact, one child was able to associate what he had learned about lead paint poisoning at the museum with his personal experience with lead poisoning at his school years before. However, the child only saw the acute effects of poisoning (likening it to a cold) and did not really understand the long-term effects of the poisoning on his health and well-being. Similarly, another child described how common it was for his neighbors to litter. He reflected upon his understanding of how litter could also pollute the air and that polluted air might make it harder for people to breathe (Figure 5). When the research staff attempted to encourage further discussions about the long-term effects of littering and environmental exposures in general on their health and how healthy eating might help to mitigate these effects, aside from a single discussion of smoking and air pollution on children with asthma, most PhotoVoice participants did not consider either to be a real concern for their own future health. 

This led to a discussion about why people litter (lack of community pride, laziness, thinking others would clean it up, no trash can nearby, *etc.*) and how children might make a difference in limiting litter both personally and as a community. When asked “What can we do about it?”, most kids understood that even at their young age (8–13 years old), they could reduce, reuse or recycle to improve the environment, but none of the children or their families actually reported participating in reduction or recycling programs or felt motivated to do so prior to the project. Several ideas about how future litter awareness and prevention education could be incorporated into the center’s summer camp and after school program. Also, two of the older participants felt that other center participants and possibly the community could be surveyed to get a better idea of why people litter in their neighborhood. None of the children had heard of the organization “Keep Cincinnati Beautiful”, an affiliate of “Keep America Beautiful” but most thought that they might be a good partner to help the center staff (albeit not them personally) implement litter programming at the center. 

### 3.7. Photo Presentation and Viewings 

Following the project, participants first made hand illustrated posters upon which to display their photos and captions. One by one, each of the six participants present were given the opportunity to present their photo and read their caption to a group of approximately 22 researchers (traditionally trained basic scientists, epidemiologists, and clinical staff) and center staffers at a holiday gift-giving event. While many enjoyed the idea of having their work seen, half of the participants asked study staff to read their captions for them, possibly because most had not ever presented to a group of adults before. Afterwards, the children indicated how excited they were to learn that others outside their community cared about them. On the receiving end, presentation attendees became more convinced of the importance of, and therefore more supportive of, the community partnership with the center. 

Additional viewings were planned. For example, ten of the photographs were selected together by participants and research and center staff, printed, and mounted 8 inch by 10 inch prints on 12 inch by 16 inch black corrugated boards along with their captions, and anonymously put them on display at the center’s annual teen-organized “Taste of the World Celebration”. Many of the participating children were in attendance and were able to see the responses and hear the comments of viewers. Several indicated that they felt empowered by it. That is, many were more willing to offer their opinions when asked, share with others their knowledge, and participate in a subsequent citizen youth science leadership and advocacy training program later offered at the center. 

Next, the photos were put on display at an event outside the community, a volunteer celebration dinner for a community HopeFest Health Screening and Education Fair. This provided an opportunity for possible future center supporters to learn about the issues experienced by children in the community by seeing their strengths and their challenges. Recently, three of the mounted prints were selected for display (over a five-month period) at a university art gallery promoting the use of “Art as Therapy” nearly 50 miles away. Upon their return, these framed prints will be permanently placed on display at the center. 

Finally, a two-page color newsletter that included many of the photos and captions was developed to highlight the project. Copies were then distributed to participants, their parents, and to both the wider research and center staff. In addition, the newsletter was circulated to potential program collaborators and agency supporters to provide them examples of both the need and potential of the community youth. As a result, faculty from around the University of Cincinnati have shown interest in coming together to assist and support programming and research at the center.

## 4. Discussion

The saying that “A picture is worth a thousand words” may be cliché, but it is in all likelihood, true, particularly for children. Photographs contain many meanings that are left to the eye of the beholder; therefore a camera gives a child a new and powerful perspective of the world and the ability to control how they see it. PhotoVoice participants who were frequent visitors to a community center serving predominantly low-income, African-American families have shared with the world a glimpse of how they perceive their health and their environment and given experienced researchers new to community engagement, unique insights into how best to approach, partner and build trust to study not only environmental health, but broader aspects of health as well. 

Like other youth-based PhotoVoice studies, nutrition and food was a theme identified and discussed [18,19,20]. From our participants’ photos, it is obvious that these children understand that nutrition is important to their physical health, yet they have few tools to assist them in improving it. Indeed, like other studies, low food-literacy, advertisement of and convenient access to unhealthy foods and limited access to fresh and healthy foods are real barriers for these low-income children highlighting a need for future programming and studies within this community on this topic [21,22].

Similarly, most of our PhotoVoice participants understood that having a more educated mind was important in achieving overall health and ultimately economic success in life, but their limited ability to plan tasks (and to stay on task often observed in our PhotoVoice sessions) and critically think at this age, was a real challenge. Needed are empowerment education and leadership programs similar to the Youth Empowerment Strategies (YES!) after-school program. Funded by a CBPR initiative of the Centers for Disease Control and Prevention, the program used an asset-based approach including PhotoVoice methodology to improve critical thinking and problem solving skills of youth in order to build their capacities and improve their overall health [23]. 

Also worrisome and not unique to our PhotoVoice participants were the gangs and violence in their neighborhoods [24,25,26]. Another study utilizing PhotoVoice focused on a Youth Advisory Board (YAB) made up of young adult Latino and African Americans living in another Midwestern city of Detroit, Michigan. Participants of this study also underscored the causes of violence including the drug trade, economic insecurity, and abandoned spaces [27]. Similar to our study, however, the report highlighted the critical roles of community centers and other community organizations (*i.e.*, museums, churches, health care and social service agencies). Community capacity building and the ability to find resources through community organizations was also one of five critical factors identified by a group of African American teens and adults to be associated with health and well-being of residents living in poverty [28]. 

Just as important, our PhotoVoice participants pointed to their relationships with friends and family as strengths in their lives. Consistent with this finding, the above mentioned study concluded that social cohesion and strong bonds with their geographic neighborhoods, as well as “within community” interactions (*i.e.*, via recreation or community gatherings), and the presence of collective group efforts to improve their community were also considered critical health promoting factors [28]. 

Like trustworthy family and friends, our participants valued a clean environment. Other PhotoVoice projects have reported the desire of cleanliness and the negative roles of trash and contemptible built environments on their communities [24,29]. While one of our older PhotoVoice participants pointed out that high levels of waste and pollution indicated a lack of pride in their neighborhood, garbage (from litter to a large discarded couch in the alley way) was hardly ever photographed, especially by the younger participants. Moreover, other types of pollution (*i.e.*, water or air pollution) were barely mentioned. It is unclear if the reason for this is that our study population included younger children or if the children simply had grown accustomed to litter and pollution. A report by the non-profit Keep America Beautiful indicated that people litter where they feel no ownership, where trash has already accumulated and where they feel others will clean up after them [30]. We speculate that without an effort to educate or provide pollution prevention programs, our PhotoVoice participants too will continue to add to the excess waste in their community. 

Also noteworthy is the observation that our participants did not seem to adequately understand how litter and pollution affected their health over their lifetimes. Unbeknown to them, the risk of chronic exposure, along with chronic stress, likely makes them more susceptible to obesity and other chronic diseases. As a result of these observations, the Citizen Youth Scientist (CYS) program has been initiated at the center. Initially, ten teen participants (including three of our pervious PhotoVoice participants) discussed more deeply the dangers of environmental exposures and how to counteract their effects. In addition, participants received training in leadership, community advocacy, and research methodology so that they can be directly involved in the planning, data collection, interpretation and dissemination of future studies at the center [31]. The program utilized in part, the Building Trust curriculum at the Maryland Center for Health Equity [32]. Current plans are to expand the program to include 20 teens and to help the larger CYS group complete an environmental audit that will use both informal interviews and geographical information system software to allow visual demonstration of both the neighborhood’s needs and assets [33]. This expansion will be supported by a new partnership with students and faculty at the University of Cincinnati and facilitated by faculty and staff at Cincinnati Children’s Hospital Medical Center.

Indeed, sharing the photos and captions with the center staff and larger center clientele via viewings and the newsletter had built significant trust and lead to a wish to expand efforts across many disciplines. Sharing our findings with the broader local and academic communities has increased support for and brought interest in collaboration on future projects that will promote health and broadly tackle health inequities. Importantly, rather than studying the individual parts of the system that influence the root causes of problems experienced by community or merely responding to their symptoms, partnership with other academic and community groups will allow us to better utilize a systems thinking approach that will assist, strengthen, and empowers residents and produce relevant and novel scientificdiscoveries [34]. Faculty across the University from the College of Medicine, Allied Health Sciences, Nursing, Design, and Business are contributors. Together, the larger group along with faculty from the University’s Center for Service Learning and Civic Engagement, may serve as a mutually beneficial community “collaboratory” that also provides an infrastructure for transformative educational, service learning, and volunteer opportunities to students, teachers and professionals alike seeking to enrich scholarship and grow academically through deeper community engagement and inclusion of reflective and interactive experiential learning. To this end, at this time, the board and staff of our partnering community center are working on a strategic plan that will incorporate these partnerships and work toward a vision of becoming a learning community that will help children and families living in the neighborhood and beyond to better seek and attain health and prosperity.

## 5. Conclusions 

With the guidance and support of seasoned qualitative researchers, PhotoVoice is an excellent approach to understand the lived experiences of and sustainably partner with community members of at-risk communities dealing with complex environmental health issues. Overall, as an inexpensive needs and assets assessment, PhotoVoice helped us to identify and has provided preliminary data for future health education, health promotion and intervention programs and research studies specifically suited to this unique population. It has also highlighted a need to better educate children and their families regarding the health effects of the environment and provide them relevant tools (*i.e.*, nutrition and lifestyle changes) and health screenings to mitigate its effects. Just as important, the study has made even more obvious the great need for systemic, structural and policy changes at all levels and invited opportunities to broadly partner with other community agencies and academics to initiate these changes while providing needed civic training to students, community advocates and researchers. 
